# Mutational landscape reflects the biological continuum of plasma cell dyscrasias

**DOI:** 10.1038/bcj.2017.19

**Published:** 2017-02-24

**Authors:** A Rossi, M Voigtlaender, S Janjetovic, B Thiele, M Alawi, M März, A Brandt, T Hansen, J Radloff, G Schön, U Hegenbart, S Schönland, C Langer, C Bokemeyer, M Binder

**Affiliations:** 1 Department of Oncology, Hematology and Bone Marrow Transplantation with Section Pneumology, Hubertus Wald Tumorzentrum/University Cancer Center Hamburg, University Medical Center Hamburg-Eppendorf, Hamburg, Germany; 2 Department of Pharmacy and Biotechnology, Alma Mater Studiorum, University of Bologna, Bologna, Italy; 3 Bioinformatics Core, University Medical Center Hamburg-Eppendorf, Hamburg, Germany; 4 Heinrich Pette Institute, Leibniz Institute for Experimental Virology, Hamburg, Germany; 5 Department of Medical Biometry and Epidemiology, University Medical Center Hamburg-Eppendorf, Hamburg, Germany; 6 Amyloidosis Center, Department of Internal Medicine, Division of Hematology/Oncology/Rheumatology, University of Heidelberg, Heidelberg, Germany; 7 Department of Internal Medicine III, University Hospital of Ulm, Ulm, Germany

## Abstract

We subjected 90 patients covering a biological spectrum of plasma cell dyscrasias (monoclonal gammopathy of undetermined significance (MGUS), amyloid light-chain (AL) amyloidosis and multiple myeloma) to next-generation sequencing (NGS) gene panel analysis on unsorted bone marrow. A total of 64 different mutations in 8 genes were identified in this cohort. *NRAS* (28.1%), *KRAS* (21.3%), *TP53* (19.5%), *BRAF* (19.1%) and *CCND1* (8.9%) were the most commonly mutated genes in all patients. Patients with non-myeloma plasma cell dyscrasias showed a significantly lower mutational load than myeloma patients (0.91±0.30 vs 2.07±0.29 mutations per case, *P*=0.008). *KRAS* and *NRAS* exon 3 mutations were significantly associated with the myeloma cohort compared with non-myeloma plasma cell dyscrasias (odds ratio (OR) 9.87, 95% confidence interval (CI) 1.07–90.72, *P*=0.043 and OR 7.03, 95% CI 1.49–33.26, *P*=0.014). *NRAS* exon 3 and *TP53* exon 6 mutations were significantly associated with del17p cytogenetics (OR 0.12, 95% CI 0.02–0.87, *P*=0.036 and OR 0.05, 95% CI 0.01–0.54, *P*=0.013). Our data show that the mutational landscape reflects the biological continuum of plasma cell dyscrasias from a low-complexity mutational pattern in MGUS and AL amyloidosis to a high-complexity pattern in multiple myeloma. Our targeted NGS approach allows resource-efficient, sensitive and scalable mutation analysis for prognostic, predictive or therapeutic purposes.

## Introduction

Plasma cell dyscrasias arise from clonal plasma cell expansions most commonly in the bone marrow (BM) and are characterized by a patient-specific monoclonal antibody or light chain, the so-called paraprotein that can be detected in the plasma of most patients. The most common plasma cell dyscrasia represents monoclonal gammopathy of undetermined significance (MGUS) that is defined as a premalignant precursor state with <10% plasma cell infiltration in the BM and absence of end-organ damage.^1^ MGUS can progress to asymptomatic or symptomatic multiple myeloma with a frequency of ∼1% per year,^2^ the latter often presenting with serious clinical problems as bone fractures, renal failure, anemia and hypercalcemia.^3^ Paraproteins may also have specific biochemical properties that interfere with correct protein folding, resulting in tissue deposition and subsequent organ damage. This is the case in systemic amyloid light-chain (AL) amyloidosis developing on the ground of light-chain dysproteinemias.^4^ Compared with other plasma cell dyscrasias, these cases are often characterized by a lower proliferative plasma cell component in the BM.^5^

Plasma cell dyscrasias are genetically heterogeneous diseases and invariably show clonal evolution over time as they progress.^6^ Translocations that place oncogenes under the strong enhancers of the IgH (immunoglobulin heavy) loci are most of the time early lesions that can also be found at the MGUS stage by fluorescent *in situ* hybridization, whereas other cytogenetic aberrancies such as del17p represent late events that are acquired in the course of the disease.^7^ Similarly, AL amyloidosis involves cytogenetically less complex plasma cells with prognostically rather favorable lesions, whereas multiple myeloma more often shows more complex and sometimes poor prognosis genetic aberrations.^8, 9, 10^

Evidence from whole-genome sequencing studies in myeloma suggests, however, that plasma cell disorders are not only driven by such cytogenetic lesions, but also by oncogenic mutations that may even more reflect their genetic heterogeneity.^11, 12^ Most of the data have been generated in patients with classical myeloma, although the mutational landscape of AL amyloidosis or MGUS still remains unexplored. In classical myeloma, mutations occur in different pathways with genes involved in RNA processing, protein translation and the unfolded protein response. Most frequently mutations were found in *NRAS*, *KRAS*, *FAM46C*, *TP53*, *BRAF*, *NFKB1*, *CYLD*, *LTB*, *IRF4* and *CCND1*.^13, 14, 15, 16^ Many of these mutations are conceived as driver mutations, some of which potentially druggable, at least if present in more than a tumor subclone, and others have prognostic relevance.^17, 18, 19, 20, 21, 22, 23^ It is therefore vital to develop clinically utilizable tools that may help to quickly generate a picture of the clonal architecture of a given patient with a plasma cell disorder.

Here we developed a targeted approach to determine a panel of recurrent oncogenic myeloma mutations with state-of-the-art technology in the biological spectrum of plasma cell disorders including MGUS, AL amyloidosis and multiple myeloma. We establish that the genetic complexity—just as the cytogenetic aberrations—closely reflects the clinical biology of these plasma cell disorders. Moreover, our PCR-based deep sequencing approach with a turnaround time of ∼3 days is attractive for routine clinical use for prognostication and identification of potentially druggable targets.

## Materials and methods

### Patient characteristics and material

BM mononuclear cells of 11 MGUS cases, 24 AL amyloidosis cases and 55 multiple myeloma cases were collected during routine diagnostic BM aspirations. All patients consented to the use of their biological material for this investigation. Myeloma-related chromosomal abnormalities were assessed by interphase fluorescence *in situ* hybridization using commercially available probes LSI *TP53* for detecting 17p deletion, and dual-color translocation probe FGFR3/IGH for detecting translocation t(4;14) (Abbott Diagnostics, Chicago, IL, USA).

### Multiplex PCR and NGS

Genomic DNA was extracted from ficollized BM by standard procedures using the NucleoSpin Tissue XS kit (Macherey-Nagel, Düren, Germany). DNA quality and quantity was assessed using a Nanodrop1000 (Thermo Fisher Scientific, Wilmington, DE, USA). To amplify informative coding regions of 10 genes (*KRAS*, *NRAS*, *FAM46C*, *TP53*, *NFKB1*, *LTB*, *IRF4*, *BRAF*, *CYLD* and *CCND1*), a multiplex PCR was set up using the Phusion HS II (Thermo Fisher Scientific). All primer pairs are shown in Supplementary Table S1. A total of 50 ng of genomic DNA was amplified per PCR. Amplicons were subjected to PCR-based barcoding, cut out from agarose gels and purified following standard procedures (NucleoSpin gel and PCR clean-up, Macherey-Nagel). Samples were pooled in an equimolar ratio and quality as well as quantity assessment was performed using a 2100 Bioanalyzer (Agilent Technologies, Santa Clara, CA, USA) and a Quibit Fluorometer (Thermo Fisher Scientific). Multiplex sequencing was performed with a 600-cycle single indexed (7 nucleotides) paired-end run on a MiSeq sequencer (Illumina, San Diego, CA, USA) at an estimated depth of 100 000 reads per sample.

### Sensitivity determination

The colon cancer cell line SW620 (ATCC, Manassas, VA, USA), harboring a *KRAS* exon 2 mutation, was used to evaluate the limit of detection of our next-generation sequencing (NGS) approach. One to 1000 genomes of this cell line were spiked into 10 000 genomes of the Colo320 cell line carrying no *KRAS* mutation (ATCC). NGS was performed as described above at an estimated depth of 20 000 reads per sample.

### NGS data analysis

An inhouse bioinformatics pipeline optimized for the diagnostic workflow was used to analyze the MiSeq data. In brief, adapter sequences and low-quality (Phred quality score <10) bases were removed from sequencing reads with Trimmomatic (v0.32).^24^ Overlapping paired reads were merged, dereplicated and clustered using USEARCH (v8.1.1831).^25^ Sequences observed <10 times were discarded after the dereplication step. BLAT^26^ was employed to align the resulting clusters to reference gene sequences. The background error rate of the sequencer together with PCR artifacts was calculated using a known single-nucleotide polymorphism in the *LTB* gene. Variants other than the known two base pairs were counted and related to the local coverage.

### Statistics

Data were presented as mean±s.e.m. Differences in the mutational load between the two cohorts of multiple myeloma and non-myeloma plasma cell dyscrasias were analyzed using the two-sided Student's *t-*test. Categorical data were compared using the χ^2^ test. Confidence intervals (CIs) in case of binomial parameter were calculated according to the Clopper–Pearson method. Multivariate logistic regression analyses with all exons mutated in ⩾5% of all patients were performed to determine mutated genes associated with disease categories, del17p and translocation t(4;14), respectively. Analyses were carried out using IBM SPSS version 22 (IBM, New York, NY, USA). A *P-*value of <0.05 was considered statistically significant.

## Results

### Patient characteristics

Targeted sequencing studies were performed on BM mononuclear cells of a cohort of 90 patients with confirmed plasma cell disorders treated and/or followed at the University Medical Center of Hamburg-Eppendorf, Ulm and Heidelberg. These included 11 MGUS, 24 AL amyloidosis and 55 multiple myeloma cases. Clinical characteristics of this cohort are summarized in Table 1.

### Targeted multiplex NGS shows high sensitivity and specificity

For sensitivity determination, a cell line with a known *KRAS* mutation was spiked at different ratios into genomic material of an unmutated cell line and sequenced as described in the Materials and methods section. NGS resulted in a linear relationship with increasing amounts of mutant DNA. The *KRAS* mutation was positively detected down to a ratio of 10 mutated in 10 000 unmutated genomes (0.1%), demonstrating a high sensitivity of this approach necessary to detect even minimal mutated subclones because of clonal heterogeneity or low plasma cell infiltration rate in unsorted BM.

Specificity determination was performed using a known single-nucleotide polymorphism in our data set as an internal reference as described. This analysis showed an error rate of 15 false nucleotides per 507 761 reads (error rate 0.003%±s.d. 0.0004).

These specificity and sensitivity tests led us to set a conservative detection threshold at 0.1%, implying that deviations from the germline sequence were classified as ‘mutations' if not identical to a known polymorphism and if present in >0.1% of reads.

### Targeted multiplex NGS detects gene mutations associated with plasma cell disorders

**Figure 1:**
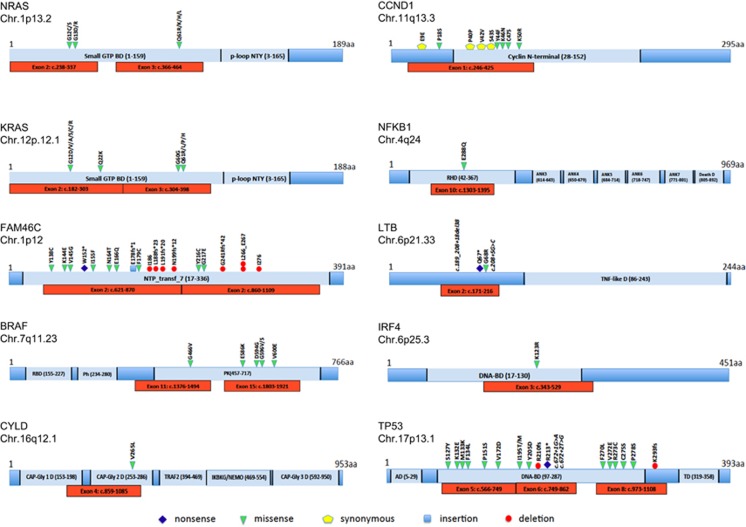
Panel of genes and hot spot regions covered by the next-generation sequencing panel including previously identified alterations. Alteration type and location of mutations in *NRAS*, *KRAS*, *FAM46C*, *CCND1*, *IRF4*, *BRAF*, *CYLD*, *TP53*, *NFKB1* and *LTB* genes previously identified in multiple myeloma are shown. Red bars indicate regions chosen for hot spot sequencing. AD, transactivation domain; ANK, ankyrin domain; BD, binding domain; CAP-Gly, cytoskeleton-associated protein glycine-rich; DAG, diacilglycerol; NTP_transf_7, nucleotidyltransferase; p-loop NTY, containing nucleoside triphosphate hydrolase; Ph, phorbol-ester/DAG-type; RBD, ras binding domain; PK, protein kinase; RHD, real like domain; TD, tetramerization domain; TNF, tumor necrosis factor domain.

A total of 10 genes covering 7 hot spots and 9 complete coding regions were chosen for this multiplex PCR NGS panel based on mutational frequencies observed in previous whole-genome studies on multiple myeloma.^13, 14^ Figure 1 gives an overview of all sequenced genes and previously identified mutational hot spot regions.

**Figure 2:**
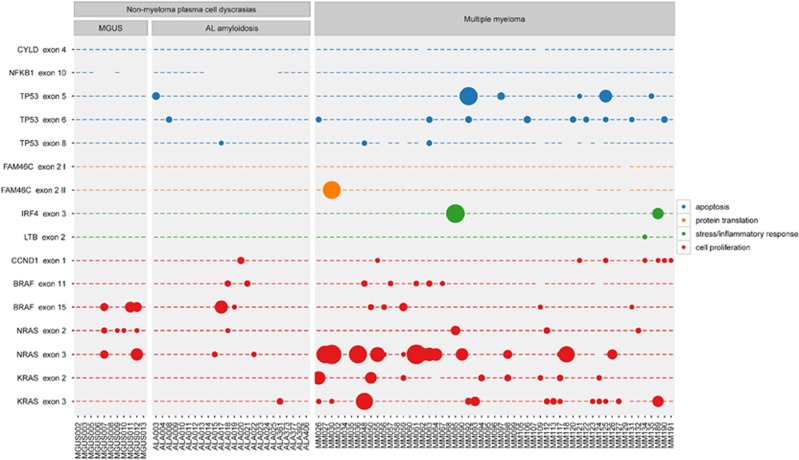
Mutated clones detected by NGS in the MGUS, AL amyloidosis and myeloma cohorts. Genes regulating cell proliferation (red circles), stress and inflammatory response (green circles), apoptosis (blue circles) and protein translation (orange circles) are shown.

All samples successfully completed targeted sequencing with a median coverage of 5727 × per amplicon. A total of 64 different mutations were detected after removal of background and nonfunctional variants as well as single-nucleotide polymorphisms (Figure 2 and Table 2). In 32 patients (35.6%), no mutations could be identified. *NRAS* mutations were most commonly found in our samples (28.1%), followed by *KRAS* (21.3%), *TP53* (19.5%), *BRAF* (19.1%) and *CCND1* (8.9%), whereas *FAM46C*, *IRF4* and *LTB* were mutated only in one to three patients. No mutations were found in the *CYLD* or *NFKB1* gene in our cohort.

### Complexity of the mutational landscape in different subsets of plasma cell dyscrasias

Comprehensive mutational profiling has been largely restricted to classical myeloma so far. Here, we set out to determine the mutational architecture of plasma cell dyscrasias with lower proliferative plasma cell components and compared it with classical myeloma.

**Figure 3:**
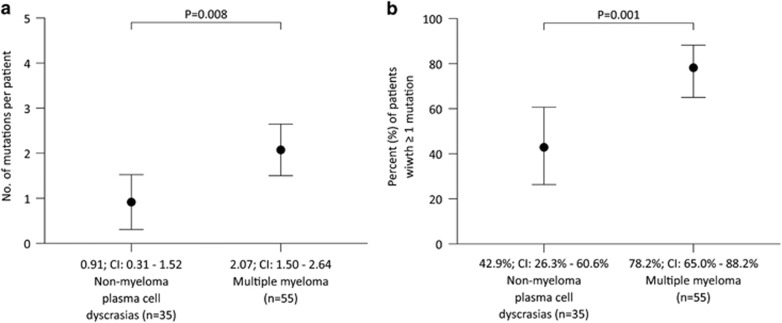
Differences in the mutational load between disease categories. (**a**) Difference in mutational frequency (number of mutant exons per patient) between myeloma and non-myeloma plasma cell dyscrasias. (**b**) Difference in percentage of patients with mutations (⩾1 mutation per case) between myeloma and non-myeloma plasma cell dyscrasias.

MGUS showed mutations only in *NRAS* (exons 2 and 3) and *BRAF* (exon 15) with a mutation frequency of 36.4% and 27.3%, respectively. AL amyloidosis revealed a frequency of mutated cases of 41.7% and these were restricted to *KRAS* (4.2%), *NRAS* (12.5%), *TP53* (12.5%), *BRAF* (16.7%) and *CCND1* (4.2%). In contrast, multiple myeloma showed a more complex mutational landscape with mutations in *KRAS* (33.3%), *NRAS* (33.3%), *BRAF* (18.5%), *TP53* (26.9%), *CCND1* (12.7%), *FAM46C* (1.9%), *IRF4* (3.6%) and *LTB* (1.8%) genes, in line with previous studies (Table 3). Overall, 78.2% of myeloma cases carried mutations in the investigated genes. We found an overlap of mutations in *KRAS* and *NRAS* genes activating mitogen-activated protein kinase signaling in 5/54 myeloma patients (9.3%), most likely in different tumor subclones because of different percentages of mutant reads. The mutational frequency (mutated amplicons per patient) was statistically different between patients with myeloma and those with non-myeloma plasma cell dyscrasias (*P*=0.008), with more mutations occurring in myeloma (2.07±0.29) compared with patients with MGUS and AL amyloidosis (0.91±0.30, Figure 3a). The same was true when comparing the numbers of patients with at least one mutation with unmutated cases (78.2% in the myeloma cohort vs 42.9% in the cohort of non-myeloma plasma cell dyscrasias, *P*=0.001, Figure 3b). In a multivariate logistic regression analysis including all exons mutated in ⩾5% of cases (*KRAS* exons 2 and 3, *NRAS* exons 2 and 3, *TP53* exons 5 and 6, *BRAF* exons 11 and 15 and *CCND1* exon 1), *KRAS* exon 3 and *NRAS* exon 3 were significantly associated with the multiple myeloma disease category compared with patients with non-myeloma plasma cell dyscrasias (odds ratio (OR) 9.87, 95% CI 1.07–90.72, *P*=0.043 and OR 7.03, 95% CI 1.49–33.26, *P*=0.014, Table 4).

### Correlation of mutational profile with conventional cytogenetics

Of all exons mutated in ⩾5% of cases, mutations on *NRAS* exon 3 and *TP53* exon 6 were significantly associated with del17p cytogenetics (OR 0.12, 95% CI 0.02–0.87, *P*=0.036 and OR 0.05, 95% CI 0.01–0.54, *P*=0.013, respectively, Table 5), whereas there were no significant associations between high-frequency mutations and a translocation t(4;14).

## Discussion

Whole-genome studies reveal an evolving mutational landscape that not only refines our view on the molecular drivers underlying plasma cell proliferation, but also adds a new prognostic and also therapeutic dimension.^11, 32, 33^ Here, we set out to establish such a panel for targeted NGS on an Illumina MiSeq platform. Therefore, we identified the most frequently mutated genes and hot spot regions in multiple myeloma, set up a multiplex PCR-based amplification strategy and tested this panel on unsorted BM samples of a cohort of 90 patients covering a range of plasma cell disorders. Our approach proofed to have a high sensitivity and specificity as well as a turnaround time of ∼3 days including data analysis, making it suitable for clinical application. The major strength of this approach consists in the fact that it requires only basic knowledge of primer design and evaluation of multiplex PCR and that it may conveniently be adapted to special clinical and research interests as new potentially interesting targets—also those involved in resistance—emerge.

From a biological perspective, our data set reveals interesting aspects concerning the mutational landscape of a range of plasma cell disorders that have not been covered in previous whole-genome or targeted sequencing studies to date. Interestingly, we found—comparable to conventional cytogenetics—that the mutational landscape closely reflects the biological spectrum of these conditions, from dyscrasias with a low proliferative plasma cell component like MGUS or AL amyloidosis to multiple myeloma with higher proliferative potential. The sensitivity threshold for mutation detection of 0.1% and the sequencing depth of 100 000 reads per sample rendered our approach suitable even for conditions with a low BM infiltration rate, as with a PCR input of 50 ng we were able to pick up all mutations per 7500 BM cells. Although working with whole BM instead of sorted plasma cells may have disadvantages related to more difficult clonality/subclonality determination, it is in our view the more suitable approach when comparing the clonal architecture of conditions with differing degrees of BM infiltration (42.7% mean BM infiltration in our myeloma cohort vs 20.6% in AL amyloidosis and <10% in MGUS). This is because our approach normalizes the number of mutated amplicons to a constant number of BM cells instead of an artificially enriched plasma cell population. Therefore, our numbers more linearly reflect the mutational burden of the whole tumor mass.

The depth of sequencing of our study is higher than in the ones previously reported and this allows for a validation of numerous low burden variants and provides enough resolution to dissect the subclones of the tumor. Concerning the *TP53* gene, we detected mutations in 26.9% of our myeloma patients. In accordance with Lodé *et al.*^28^ and other more recent papers, most of the mutations identified here were single-nucleotide missense mutations.^12, 13, 15^ We observed a higher frequency of mutations with respect to Lionetti *et al.*^29^ and Walker *et al.*,^15^ a finding that can be related to the higher coverage of our targeted NGS approach. Moreover, *TP53* mutations were significantly correlated with del17p cytogenetics, consistent with the literature.^13^ In line with previous studies, we report a high number of mutations in the mitogen-activated protein kinase signaling pathway with many, most often subclonal mutations in *NRAS*, *KRAS* and *BRAF.*^13, 27^ This suggests a striking subclonal convergence on this pathway in myeloma that may be exploited therapeutically. The fact that our panel includes prognostically relevant genes (*NRAS*, *KRAS*, *TP53*, *BRAF*) as well as potentially actionable targets or pathways (*RAS*, *TP53*, *BRAF*, *CCND1*, *IRF4*) also renders our approach a useful tool for improving prognostication and treatment in plasma cell disorders.^17, 18, 19, 20, 21, 22, 23^ The complex genomic architecture evident in our data set, however, highlights the need for therapeutic strategies directed at multiple targets rather than at a single genomic anomaly and underscores the success of combination therapies.

Taken together, we characterized the mutational landscape of a patient cohort with plasma cell dyscrasias using an NGS-based approach that may easily be adapted to other clinical or scientific contexts. Future technical modifications of this platform should integrate translocation detection and add more targets involved in drug resistance to ultimately track clonal variability, more precisely predict prognosis and guide treatment decisions with one simple assay in clinical routine diagnostics.

## Figures and Tables

**Table 1:** Baseline characteristics of all 90 patients

	*MGUS (*n=*11)*	*AL amyloidosis (*n=*24)*	*Multiple myeloma (*n=*55)*
Female, no. (%)	5 (45.5%)	11 (45.8%)	17 (30.9%)
Age in years, mean±s.e.m.	68.4±2.92	62.5±2.74	65.4±1.44
del17p, no. (%)	0 (0%)	2/22 (9%)	7/40 (17.5%)
Translocation t(4;14), no. (%)	0 (0%)	1/22 (4.5%)	6/38 (15.8%)

*Subtype, no. (%)*			
IgG kappa	2/11 (18.2%)		16/49 (32.7%)
IgG lambda		2/23 (8.7%)	10/49 (20.4%)
IgA kappa	1/11 (9.1%)		12/49 (24.5%)
IgA lambda			4/49 (8.2%)
Kappa light chain	5/11 (45.5%)	7/23 (30.4%)	4/49 (8.2%)
Lambda light chain	2/11 (18.2%)	14/23 (60.9%)	3/49 (6.1%)
Biclonal light chain	1/11 (9.1%)		
BM infiltration (%), mean±s.e.m.	<10	20.6±4.6	42.7±4.12

*ISS, no. (%)*			
I			15/42 (35.7%)
II			11/42 (26.2%)
III			16/42 (38.1%)

*Setting at BM sampling, no. (%)*			
First diagnosis			40/55 (72.7%)
Relapse			15/55 (27.3%)

Abbreviations: AL amyloidosis, amyloid light-chain amyloidosis; BM, bone marrow; del17p, 17p deletion; ISS International Staging System; MGUS, monoclonal gammopathy of undetermined significance.

**Table 2:** Description of the genes and type of mutations identified by NGS in the present data set

		*Variant*	*AA change*	*Mutation*	*Cancer*	*COSMIC*	*MM literature*	*No. of patients*
*NRAS*	1	c.34G>T	p.G12C	Missense	MM^a^	/	^Refs. 11, 12, 27^	4
	2	c.38G>A	p.G13D	Missense	MM	/	^Ref. 12^	1
	3	c.37G>C	p.G13R	Missense	MM	/	^Refs. 13, 14, 16, 27^	2
	4	c.37G>T	p.G13C	Missense	MM	/	MMRF	1
	5	c.38G>T	p.G13V	Missense	HL, S, LI, CN, ST	COSM574	/	1
	6	c.145G>A	p.E49K	Missense	L,S	COSM14199	/	5
	7	c.182A>G	p.Q61R	Missense	MM	/	^Refs. 11, 12, 13, 16, 27^	5
	8	c.181C>A	p.Q61K	Missense	MM	/	^Refs. 11, 13, 16, 27^	7
	9	c.182A>T	p.Q61L	Missense	MM	/	^Ref. 12, 27^	2
	10	c.190T>G	p.Y64D	Missense	MM	/	^Ref. 15^	1
*KRAS*	11	c.35G>A	p.G12D	Missense	MM	/	^ref. 12, 15, 27^	2
	12	c.35G>C	p.G12A	Missense	MM	/	^Refs. 12, 13, 14, 27^	1
	13	c.34G>C	p.G12R	Missense	MM	/	^Refs. 11, 13, 14, 16, 27^	1
	14	c.34G>A	p.G12S	Missense	MM	/	^Ref. 27^	2
	15	c.35G>T	p.G12V	Missense	MM	/	^Refs. 12, 13, 27^	1
	16	c.38G>A	p.G13D	Missense	MM	/	^Refs. 11, 12, 13, 16, 27^	2
	17	c.73C>T	p.Q25*	Nonsense	LI	COSM5352251	/	4
	18	c.109G>A	p.E37K	Missense	HL, LI, L, P, BT	COSM3738516	/	2
	19	c.169G>A	p.D57N	Missense	LI	COSM1166779	/	2
	20	c.182A>G	p.Q61R	Missense	MM	/	^Refs. 11, 13, 16, 27^	2
	21	c.182A>C	p.Q61P	Missense	LI	COSM551	/	4
	22	c.183A>T	p.Q61H	Missense	MM	/	^Refs. 11, 13, 16, 27^	7
	23	c.181C>A	p.Q61K	Missense	MM	/	^Ref. 13^	1
	24	c.201G>A	p.M67I	Missense	MM	/	^Ref. 11^	1
*FAM46C*	25	c.824_826del	p.I276delI	In-frame_D	MM	/	^Ref. 13^	1
*TP53*	26	c.376T>G	p.Y126D	Missense	MM^a^	/	^Refs. 12, 28^	1
	27	c.390_392del	p.N131delN	In-frame_D	LI, LV, HL	COSM4968986	/	1
	28	c.415A>G	p.K139R	Missense	K, B	COSM45063	/	1
	29	c.437G>A	p.W146*	Nonsense	O, P, LV, S	COSM43609	/	1
	30	c.440T>G	p.V147G	Missense	HL	COSM44309	/	1
	31	c.520A>G	p.R174G	Missense	P	COSM43763	/	1
	32	c.538G>A	p.E180K	Missense	PLC	/	^Ref. 29^	1
	33	c.558T>A	p.D186E	Missense	UAT	COSM45637	/	2
	34	c.569C>T	p.P190L	Missense	MM	/	^Ref. 28^	1
	35	c.574C>T	p.Q192*	Nonsense	O, UAT, L, LV, P	COSM19733	/	5
	36	c.587G>T	p.R196L	Missense	MM^a^	/	^Refs. 12, 16, 30^	1
	37	c.587G>A	p.R196Q	Missense	ST,B,Th	COSM44599	/	2
	38	c.589G>A	p.V197M	Missense	UAT, P	COSM43779	/	1
	39	c.637C>G	p.R213G	Missense	MM^a^	/	^Ref. 15^	1
	40	c.638G>A	p.R213Q	Missense	MM^a^	/	^Ref. 15^	4
	41	c.637C>T	p.R213*	Nonsense	MM	/	^Ref. 15^	3
	42	c.646G>A	p.V216M	Missense	MM	/	MMRF	1
	43	c.647T>G	p.V216G	Missense	UAT, O, E, LI, S	COSM43681	/	1
	44	c.661G>A	p.E221K	Missense	SG, V	COSM44853	/	3
	45	c.670G>A	p.E224K	Missense	HL, L, LI, UT	COSM10894	/	1
	46	c.892G>A	p.E298K	Missense	HL, ED	COSM44031	/	3
*BRAF*	47	c.1324G>A	p.G442S	Missense	S	COSM253323	/	1
	48	c.1331G>A	p.R444Q	Missense	ED	COSM21601	/	1
	49	c.1345G>A	p.D449N	Missense	B	COSM3832071	/	7
	50	c.1349G>A	p.W450*	Nonsense	S	COSM253324	/	1
	51	c.1363G>A	p.G455R	Missense	S	COSM1162151	/	1
	52	c.1390G>A	p.G464R	Missense	MM^a^	/	^Ref. 15^	2
	53	c.1396G>A	p.G466R	Missense	MM^a^	/	^Ref. 15^	2
	54	c.1400C>T	p.S467L	Missense	MM	/	^Ref. 15^	1
	55	c.1405G>A	p.G469R	Missense	MM^a^	/	^Refs. 16, 27^	2
	56	c.1756G>A	p.E586K	Missense	MM	/	^Ref. 13^	2
	57	c.1780G>A	p.D594N	Missense	MM	/	^Ref. 27^	1
	58	c.1790T>G	p.L597R	Missense	MM^a^	/	^Ref. 12^	1
	59	c.1799T>A	p.V600E	Missense	MM	/	^Refs. 13, 15, 16, 27^	3
	60	c.1807C>T	p.R603*	Nonsense	St, En	COSM33729	/	1
	61	c.1843G>A	p.G615R	Missense	S	COSM1140	/	2
*CCND1*	62	c.122C>T	p.S41L	Missense	UT	COSM415762	/	8
*LTB*	63	c.202G>C	p.G68R	Missense	MM	/	^Ref. 15^	1
*IRF4*	64	c.368A>G	p.K123R	Missense	MM	/	^Refs. 15, 16, 31^	2

Abbreviations: AA, amino acid; B, breast; BT, biliary tract; CN, central nervous system; E, esophagus; ED, endometrium; En, endometrium; HL, hematopoietic and lymphoid; K, kidney; L, lung; LI, large intestine; LV; liver; MM, multiple myeloma; MMRF, Multiple Myeloma Research Foundation; NGS, next-generation sequencing; O, ovary; P, pancreas; PLC, plasma cell leukemia; S, skin; SG, salivary gland; St, stomach; ST, soft tissue; T, thyroid; Th, thymus; UAT, upper aerodigestive tract; UT, urinary tract; V, vulva.

a Different amino acid substitution as previously reported.

**Table 3:** Review of the literature

	*Our data set*	*MM literature*	*Sequencing methodology*	*Material*	*Sequencing machine*	*References*
	*% Frequency*	*% Frequency*				
*NRAS*	33.3	18	Library prep.	Sorted BM	GA-II Illumina	^11^
		20	Library prep.	Sorted BM	GA-II or HiSeq Illumina	^12^
		25	Library prep.	Sorted BM	HiSeq Illumina	^13^
		20.8	PCR ampl.	Sorted BM	PGM Life Technologies	^14^
		19.4	Library prep.	Sorted BM	GA IIX Illumina	^15^
		23.7	Library prep.	Sorted BM	GA-II Illumina	^16^
		26.5	PCR ampl.	Sorted BM	Genome Seq. Junior (Roche)	^27^
*KRAS*	33.3	31.8	Library prep.	Sorted BM	GA-II Illumina	^11^
		23	Library prep.	Sorted BM	GA-II or HiSeq Illumina	^12^
		25	Library prep.	Sorted BM	HiSeq Illumina	^13^
		13.9	PCR ampl.	Sorted BM	PGM Life Technologies	^14^
		21.2	Library prep.	Sorted BM	GA IIX Illumina	^15^
		26.3	Library prep.	Sorted BM	GA-II Illumina	^16^
		32.6	PCR ampl.	Sorted BM	Genome Seq. Junior (Roche)	^27^
*FAM46C*	1.9	11	Library prep.	Sorted BM	GA-II or HiSeq Illumina	^12^
		12	Library prep.	Sorted BM	HiSeq Illumina	^13^
		5.6	Library prep.	Sorted BM	GA IIX Illumina	^15^
		13	Library prep.	Sorted BM	GA-II Illumina	^16^
*TP53*	26.9	8	Library prep.	Sorted BM	GA-II or HiSeq Illumina	^12^
		15	Library prep.	Sorted BM	HiSeq Illumina	^13^
		27.8	PCR ampl.	Sorted BM	PGM Life Technologies	^14^
		11	Library prep.	Sorted BM	GA IIX Illumina	^15^
		8	Library prep.	Sorted BM	GA-II Illumina	^16^
		3	PCR ampl.	Sorted BM	Genome Seq. Junior (Roche)	^29^
*BRAF*	18.5	6	Library prep.	Sorted BM	GA-II or HiSeq Illumina	^12^
		15	Library prep.	Sorted BM	HiSeq Illumina	^13^
		4.2	PCR ampl.	Sorted BM	PGM Life Technologies	^14^
		6.7	Library prep.	Sorted BM	GA IIX Illumina	^15^
		4	Library prep.	Sorted BM	GA-II Illumina	^16^
		10.6	PCR ampl.	Sorted BM	Genome Seq. Junior (Roche)	^27^
*CCND1*	12.7	3	Library prep.	Sorted BM	HiSeq Illumina	^13^
		1.4	PCR ampl.	Sorted BM	PGM Life Technologies	^14^
		5	Library prep.	Sorted BM	GA-II Illumina	^16^
*LTB*	1.8	3	Library prep.	Sorted BM	GA IIX Illumina	^15^
*IRF4*	3.6	3.2	Library prep.	Sorted BM	GA IIX Illumina	^15^
		2	Library prep.	Sorted BM	GA-II Illumina	^16^
		4	Library prep.	Sorted BM	GA-II Illumina	^31^
*CYLD*	0	2	Library prep.	Sorted BM	GA-II or HiSeq Illumina	^12^
		3	Library prep.	Sorted BM	HiSeq Illumina	^13^
		4.2	PCR ampl.	Sorted BM	PGM Life Technologies	^14^
		2.4	Library prep.	Sorted BM	GA IIX Illumina	^15^
*NFKB1*	0	1.5	Library prep.	Sorted BM	HiSeq Illumina	^13^

Abbreviations: ampl, amplification; BM, bone marrow; GA, Genome Analyzer; MM, multiple myeloma; prep, preparation.

**Table 4:** Association between frequently mutated genes and the ‘multiple myeloma' disease category (vs non-myeloma plasma cell dyscrasias)^a^

	*Odds ratio (95% CI)*	P-*value*
*KRAS* exon 2	b	0.999
***KRAS*** **exon 3**	**9.87 (1.07–90.72)**	**0.043**
*NRAS* exon 2	0.67 (0.12**–**3.72)	0.644
***NRAS*** **exon 3**	**7.03 (1.49–33.26)**	**0.014**
*TP53* exon 5	4.38 (0.41**–**47.44)	0.224
*TP53* exon 6	8.98 (0.86**–**94.09)	0.067
*BRAF* exon 11	3.10 (0.48**–**19.95)	0.235
*BRAF* exon 15	0.17 (0.02**–**1.56)	0.118
*CCND1* exon 1	5.03 (0.50**–**50.51)	0.170

Abbreviation: 95% CI, 95% confidence interval.

a All exons mutated in ⩾5% of all patients were included in the multivariate logistic regression analysis. Exons were counted as mutated if ⩾1 mutation was present.

b Cannot be estimated as there was no patient with ⩾1 KRAS exon 2 mutation in the cohort with non-myeloma plasma cell dyscrasias. Statistical significant values are highlighted in bold.

**Table 5:** Association between frequently mutated genes and evidence of del17p^a^

	*Odds ratio (95% CI)*	P*-value*
*KRAS* exon 2	1.15 (0.07–19.13)	0.921
*KRAS* exon 3	0.40 (0.04–3.59)	0.409
*NRAS* exon 2	b	0.999
***NRAS*** **exon 3**	**0.12 (0.02–0.87)**	**0.036**
*TP53* exon 5	1.07 (0.04–33.18)	0.968
***TP53*** **exon 6**	**0.05 (0.01–0.54)**	**0.013**
*BRAF* exon 11	0.38 (0.02–6.82)	0.515
*BRAF* exon 15	0.97 (0.05–20.13)	0.985
*CCND1* exon 1	1.29 (0.47–35.21)	0.882

Abbreviation: 95% CI, 95% confidence interval.

a All exons mutated in ⩾5% of all patients were included in the multivariate logistic regression analysis. Exons were counted as mutated if ⩾1 mutation was present.

b Cannot be estimated as there was no patient with ⩾1 NRAS exon 2 mutation in the cohort of patients with del17p. Statistical significant values are highlighted in bold.
