# Supplementary material for: Mutational landscape reflects the biological continuum of plasma cell dyscrasias
Source: Blood Cancer J. 2017 Feb 24;7(2):e537–. doi: 10.1038/bcj.2017.19 (PMC5386334; doi:10.1038/bcj.2017.19)
Supplement: Supplementary Table S1 [file bcj201719x1.docx]

| **Table S1.** Illumina-sequencing primers* used for multiplex PCR amplification of mutational hotspot regions in ten myeloma associated genes | | |
| --- | --- | --- |
|  | primer name | primer sequence 5' - 3' |
|  |  |  |
| 1^st^ PCR | NRAS_exon2_fw | ACACTCTTTCCCTACACGACGCTCTTCCGATCTgttcttgctggtgtgaaatgac |
|  | NRAS_exon2_rv | TGACTGGAGTTCAGACGTGTGCTCTTCCGATCTaagtggttctggattagctgga |
|  | NRAS_exon3_fw | ACACTCTTTCCCTACACGACGCTCTTCCGATCTattgaacttccctccctccc |
|  | NRAS_exon3_rv | TGACTGGAGTTCAGACGTGTGCTCTTCCGATCTttggtctctcatggcactgt |
|  | FAM46C_exon2_I_fw | ACACTCTTTCCCTACACGACGCTCTTCCGATCTacaagctcaaaatcagtccagt |
|  | FAM46C_exon2_I_rv | TGACTGGAGTTCAGACGTGTGCTCTTCCGATCTgggtggaagtgctcagagat |
|  | FAM46C_exon2_II_fw | ACACTCTTTCCCTACACGACGCTCTTCCGATCTcacttccaccccaccgtg |
|  | FAM46C_exon2_II_rv | TGACTGGAGTTCAGACGTGTGCTCTTCCGATCTacttcctctgctgttcaagg |
|  | TP53_exon5_fw | ACACTCTTTCCCTACACGACGCTCTTCCGATCTactctgtctccttcctcttcc |
|  | TP53_exon5_rv | TGACTGGAGTTCAGACGTGTGCTCTTCCGATCTaaccagccctgtcgtctc |
|  | TP53_exon6_fw | ACACTCTTTCCCTACACGACGCTCTTCCGATCTggcctctgattcctcactga |
|  | TP53_exon6_rv | TGACTGGAGTTCAGACGTGTGCTCTTCCGATCTcagagaccccagttgcaaac |
|  | TP53_exon8_fw | ACACTCTTTCCCTACACGACGCTCTTCCGATCTttgggagtagatggagcctg |
|  | TP53_exon8_rv | TGACTGGAGTTCAGACGTGTGCTCTTCCGATCTgcttcttgtcctgcttgctt |
|  | BRAF_exon11_fw | ACACTCTTTCCCTACACGACGCTCTTCCGATCTtctgtttggcttgacttgact |
|  | BRAF_exon11_rv | TGACTGGAGTTCAGACGTGTGCTCTTCCGATCTgacttgtcacaatgtcaccac |
|  | BRAF_exon15_fw | ACACTCTTTCCCTACACGACGCTCTTCCGATCTtttcctttacttactacacctcaga |
|  | BRAF_exon15_rv | TGACTGGAGTTCAGACGTGTGCTCTTCCGATCTagcctcaattcttaccatccaca |
|  | CYLD_exon3_fw | ACACTCTTTCCCTACACGACGCTCTTCCGATCTgcgtgtttgttgcattggac |
|  | CYLD_exon3_rv | TGACTGGAGTTCAGACGTGTGCTCTTCCGATCTaattttcttaccatgtccacacc |
|  | CCND1_exon1_fw | ACACTCTTTCCCTACACGACGCTCTTCCGATCTccagctgcccaggaagag |
|  | CCND1_exon1_rv | TGACTGGAGTTCAGACGTGTGCTCTTCCGATCTgcgacgatcttccgcatg |
|  | NFKB1_exon10_fw | ACACTCTTTCCCTACACGACGCTCTTCCGATCTtgcagatgacatccagattcg |
|  | NFKB1_exon10_rv | TGACTGGAGTTCAGACGTGTGCTCTTCCGATCTatccctcaagcccaacagtt |
|  | LTB_exon2_fw | ACACTCTTTCCCTACACGACGCTCTTCCGATCTctttcagaggggtaggggtg |
|  | LTB_exon2_rv | TGACTGGAGTTCAGACGTGTGCTCTTCCGATCTggagcctggattcctagagg |
|  | IRF4_exon3_fw | ACACTCTTTCCCTACACGACGCTCTTCCGATCTtctcttcattctttcccaccag |
|  | IRF4_exon3_rv | TGACTGGAGTTCAGACGTGTGCTCTTCCGATCTgacccaaattcaggagagcc |
|  | KRAS_exon2_fw | ACACTCTTTCCCTACACGACGCTCTTCCGATCTgcctgctgaaaatgactgaa |
|  | KRAS_exon2_rv | TGACTGGAGTTCAGACGTGTGCTCTTCCGATCTagaatggtcctgcaccagtaa |
|  | KRAS_exon3_fw | ACACTCTTTCCCTACACGACGCTCTTCCGATCTccagactgtgtttctcccttc |
|  | KRAS_exon3_rv | TGACTGGAGTTCAGACGTGTGCTCTTCCGATCTtccctcattgcactgtactcc |
|  |  |  |
| 2^nd^ PCR | Linker_Seq_fw | AATGATACGGCGACCACCGAGATCTACACTCTTTCCCTACACGACGCTC |
|  | Linker_bar_Seq_rv | CAAGCAGAAGACGGCATACGAGAT(N_6-7_)GTGACTGGAGTTCAGACGTGTG |
|  |  |  |
| * *Capital letters* Illumina MiSeq specific sequences, *lower case letters* exon specific sequences. | | |
